# Visualization of Protein Sorting at the *Trans*-Golgi Network and Endosomes Through Super-Resolution Imaging

**DOI:** 10.3389/fcell.2019.00181

**Published:** 2019-09-03

**Authors:** Yan Huang, Tianji Ma, Pik Ki Lau, Jinhui Wang, Teng Zhao, Shengwang Du, Michael M. T. Loy, Yusong Guo

**Affiliations:** ^1^Division of Life Science, Hong Kong University of Science and Technology, Hong Kong, China; ^2^Light Innovation Technology Limited, Hong Kong, China; ^3^Department of Chemical and Biological Engineering, Hong Kong University of Science and Technology, Hong Kong, China; ^4^Department of Physics, Hong Kong University of Science and Technology, Hong Kong, China; ^5^Hong Kong University of Science and Technology Shenzhen Research Institute, Shenzhen, China

**Keywords:** *trans*-Golgi network, cargo adaptor, sorting, clathrin, storm

## Abstract

The *trans*-Golgi network (TGN) and endosomes are essential protein sorting stations in the secretory transport pathway. Protein sorting is fundamentally a process of spatial segregation, but the spatial relationships among the proteins that constitute the sorting machinery have not been systematically analyzed at high resolution in mammalian cells. Here, using two-color STORM imaging, we show that the TGN/endosome-localized cargo adaptors, AP-1, GGA2 and epsinR, form elongated structures of over 250 nm in length at the juxta-nuclear Golgi area. Many of these structures are associated with clathrin. We found that AP-1 is spatially segregated from AP-3 and GGA2, whereas a fraction of AP-1 and GGA2 punctae are associated with epsinR. Moreover, we observed that the planar cell polarity cargo proteins, Vangl2 and Frizzled6 associate with different cargo adaptors—AP-1 and GGA2 or epsinR, respectively—when exiting the TGN. Knockdown analysis confirms the functional significance of this segregation. Our data indicates that TGN/endosome-localized cargo adaptors have distinct spatial relationships. The spatially segregated cargo adaptors GGA2 and AP-1 regulate sorting of Frizzled6 and Vangl2, respectively and spatially associated cargo adaptors can cooperatively regulate a specific sorting process.

## Introduction

The *trans*-Golgi network (TGN) and endosomes are important transport hubs in the secretory transport pathway. To ensure the fidelity of protein transport, elaborate protein sorting machineries are employed to accurately package cargo proteins into specific transport vesicles that are delivered to various downstream compartments. Defects in the cargo sorting process cause protein mis-targeting and give rise to defects in cell polarity, immunity, and regulated secretion (Guo et al., 2014).

The key players that mediate protein sorting at the TGN and endosomes include various cytosolic cargo adaptors. Once recruited onto the membranes, these cargo adaptors recognize sorting motifs in the cytosolic domains of the transmembrane cargo proteins. Some cargo adaptors subsequently recruit clathrin. Polymerized clathrin along with their associated clathrin adaptors form characteristic electron dense membrane coat structures and this process leads to a sequestration of the associated cargo proteins into coated membrane patches (Guo et al., 2014). The assembly of vesicle coat structures also promotes deformations of the lipid bilayer leading to vesicle budding.

Protein sorting is fundamentally a process of spatial segregation: cargo sorting machineries concentrate specific cargo proteins in specific membrane microdomains from which other proteins, such as resident proteins are excluded. Despite its importance, the spatial distributions of the key players that participate in the protein sorting process at the TGN and endosomes have not been systematically analyzed at high resolution in mammalian cells. Moreover, the key step in the protein sorting process, namely the association of a specific cargo molecule with a specific cargo adaptor, has not been intensively studied using high resolution fluorescence microscopy. Addressing these questions will significantly advance our understanding of how cargo sorting machineries function to package specific cargo proteins into transport vesicles at the TGN and endosomes.

Conventional fluorescence microcopy cannot achieve the resolution necessary to distinguish the spatial relationships among Golgi-localized proteins. Immunogold electron microscopy (EM) has demonstrated that the majority of the cargo adaptors, the adaptor protein complex-1 (AP-1) and GGA2, are present in separate clathrin-coated budding profiles in Drosophila Dmel2 cells (Hirst et al., 2009). Immunogold labeling also revealed that the adaptor protein complex-3 (AP-3) and AP-1 are found on distinct buds arising from endosome-associated tubules in HepG2 cells (Peden et al., 2004). This suggests that AP-1 is not present in the same vesicles as GGAs or AP-3. Although immunogold EM analysis provides details of protein localizations at fine resolution, it requires a long procedure of sample preparation. Moreover, the efficiency of immunogold labeling relies on both the antibody quality and the target protein condition in the ultra-thin section, which in practice is usually affected by intricate sample preparation (Griffiths and Hoppeler, 1986; Howell et al., 1987; D'Amico and Skarmoutsou, 2008). Therefore, immunogold labeling for transmission EM generally displays a lower efficiency than that immunostaining for confocal microscopy does in regard to the labeling efficiency. For this reason, immunogold labeling has been used for detailed subcellular localization of the target protein instead of providing a comprehensive view of the spatial distributions of adaptor and cargo protein on the whole organelle.

Here, we utilized two-color stochastic optical reconstruction microscopy (STORM) to provide a wider view of the spatial distributions of proteins that participate in the TGN/endosome sorting process at fine resolution. Two-color STORM imaging simultaneously captures both fluorescent dyes in the same camera frame to achieve a spatial resolution of 20 nm (Zhao et al., 2015). Our set up was equipped with an active sample locking mechanism that effectively abolishes drift into the x-y plane and the z-axis. This allowed the position of the sample to be stabilized to within 1 nm (Zhao et al., 2015). We found that the TGN- and endosome- localized cargo adaptors can be assembled into elongated punctate structures of over 250 nm in length at the juxta-nuclear Golgi area. We revealed that the cargo adaptors, AP-1 and GGA2, are spatially segregated and they function to mediate sorting of planar cell polarity proteins, Vangl2 and Frizzled6, respectively. These analyses indicate that spatial segregation of cargo adaptors may contribute to the protein sorting process. In contrast, another cargo adaptor, epsinR, displayed a GGA2 and an AP-1 associated pattern. Both GGA2 and epsinR regulate sorting of Frizzled6, suggesting that spatially associated cargo adaptors can cooperatively regulate sorting of a specific cargo molecule. Our super-resolution imaging studies revealed that Frizzled6 and Vangl2 are spatially associated with different clathrin adaptors, providing a direct observation of the differential cargo sorting process.

## Results

### Analysis of the Spatial Relationships Between Clathrin and TGN/endosome-localized Cargo Adaptors

**Figure 1:**
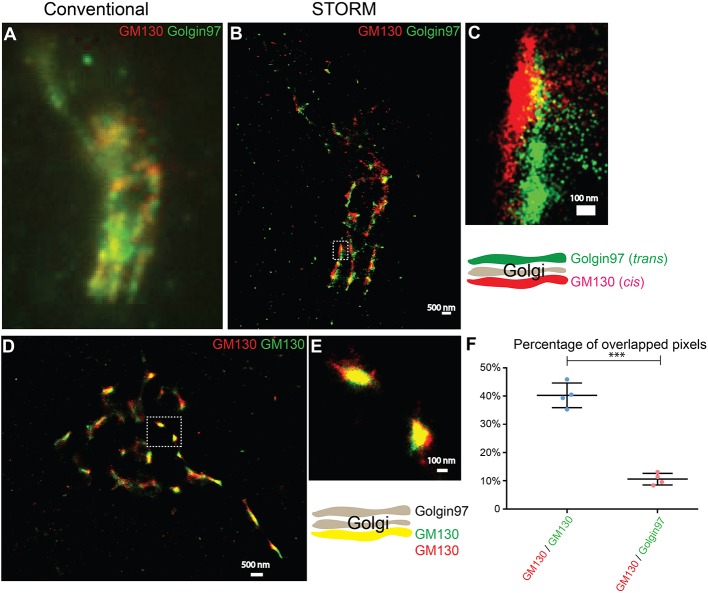
Two-color STORM can be utilized to reveal spatial relationships of Golgi-localized proteins at high resolution. **(A,B)** Localization patterns of a *cis*-Golgi marker (GM130) and a *trans*-Golgi marker (Golgin-97) in the same COS7 cell visualized through conventional fluorescent microscopy **(A)** or through two-color STORM **(B)**. Scale bar, 500 nm. **(C)** A magnified view of the indicated area in **(B)**. Scale bar, 100 nm. **(D)** Localization patterns of a *cis*-Golgi marker (GM130) labeled with two different fluorescent dyes in the COS7 cell revealed through two-color STORM. Scale bar, 500 nm. **(E)** A magnified view of the indicated area in **(D)**. Scale bar, 100 nm. **(F)** Quantification of the percentage of overlapped pixels between GM130 and Golgin-97 and between Alexa Fluor 750-labeled GM130 and Alexa Fluor 647-labeled GM130 based on four super-resolution images acquired from one experiment in each experimental group (mean ± SD). ***p < 0.001 by two-tailed Student's *t*-test. The super-resolution images showed the localization patterns of the indicated protein in the whole juxtanuclear area. Each dot in the graphs represents data of the whole juxtanuclear Golgi area of each individual cell.

To test whether two-color STORM can be used to compare the spatial relationships between different Golgi-localized proteins, we analyzed the localization patterns of a *cis*-Golgi marker, GM130, and a *trans*-Golgi marker, Golgin-97. Using conventional light microscopy, the localization pattern of GM130 was not clearly distinguishable from that of Golgin-97 (Figure 1A). By contrast, with STORM, we observed a clear separation of GM130 from Golgin-97 (Figures 1B,C). To ensure that the separation of GM130 from Golgin-97 in the super-resolution images was not due to sample drift, we analyzed the localization patterns of the same protein, GM130, labeled with two different dyes, Alexa 750 and Alexa 647. STORM images of Alexa 750 dye-labeled GM130 (GM130 ^750^) and Alexa 647 dye-labeled GM130 (GM130 ^647^) overlapped almost perfectly (Figures 1D,E). Quantification analysis indicated that the percentage of overlapping between GM130 ^750^ and GM130 ^647^ was significantly higher than that of GM130 and Golgin-97 (Figure 1F). We noticed that GM130 ^750^ and GM130 ^647^ were not completely overlapped. A possible explanation is that not all the GM130 proteins are labeled with both Alexa 647 dye and Alexa 750 dye. To ensure that this is not due to a systematic error, we performed experiments using imaging beads coated with both Alexa 750 and Alexa 647. Our results indicate that Alexa 750 and Alexa 647 showed a nearly perfectly overlapped pattern (Figures S1A–D). Thus, two-color STORM is a feasible tool to reveal the spatial relationship between different Golgi-localized proteins.

Using two-color STORM, we sought to analyze the spatial relationships between clathrin and cargo adaptors in COS7 cells. COS7 cells are flat so that the Golgi is close to the coverslips for STORM imaging. When antibodies are commercially available, we determined the localization of the endogenous proteins, including clathrin heavy chain (CHC), GGA2, the γ subunit of the adaptor complex-1 (γ1) and the δ subunit of the adaptor complex 3 (AP3δ1). We generated a FLAG-tagged version of epsinR and used antibodies against FLAG to label epsinR after transient transfection. In COS7 cells, CHC, γ1, GGA2, and FLAG are highly clustered at the juxtanuclear area where the Golgi is localized and some punctate structures in the cell periphery, presumably on endosomes (Figures S2A–L,P–R). In comparison, the structures positive for AP3δ1 were more spread out (Figures S2M–O). Similar patterns labeled were observed in HeLa cells (Figures S3A,C,E,G,I). In addition, the fluorescent intensities of the juxtanuclear structures labeled for the cargo adaptor were greatly diminished when cells were transfected with siRNAs against the corresponding protein (Figures S3B,D,F,H,J). These siRNAs effectively reduced the expression level of their target proteins (Figures S3K–M and **Figure 4Q**) (Ma et al., 2018). This result demonstrates that the signals we detected were specific. We selected the whole juxtanuclear Golgi area where the majority of the cargo adaptors were localized for the following super-resolution imaging analyses.

**Figure 2:**
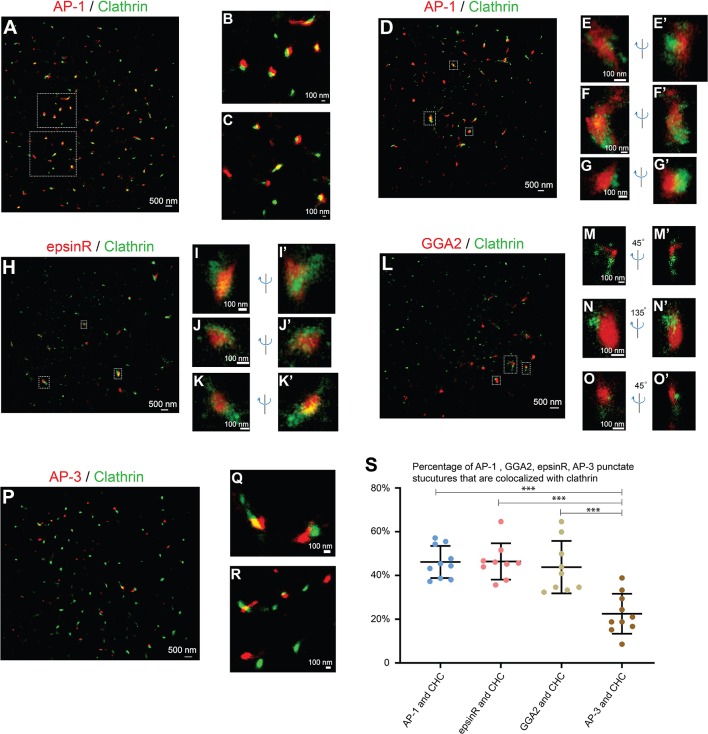
Spatial relationship analysis between cargo adaptors and clathrin at the juxtanuclear area. **(A–G')** COS7 cells were co-stained with antibodies against clathrin heavy chain and γ1-adaptin. Localization patterns of AP-1 marked by γ1-adaptin and clathrin marked by clathrin heavy chain in the same COS7 cell were analyzed by two-color STORM in 2-dimension **(A–C)** and 3-dimension **(D–G')**. Magnified view of the indicated area in **(A,D)** were shown in **(B,C)** and **(E–G')**, respectively. **(H–K')**. COS7 cells were transfected with epsinR-FLAG. Day 1 after transfection, cells were co-stained with antibodies against γ1-adaptin and FLAG tag. 3- Dimensional localization patterns of epsinR-FLAG and clathrin heavy chain in the same COS7 cell were analyzed through two-color STORM. Magnified views of the indicated area in **(H)** were shown in **(I–K')**. **(L–O')** COS7 cells were co-stained with antibodies against clathrin heavy chain and GGA2. Localization patterns of GGA2 and clathrin heavy chain in the same COS7 cell were analyzed by two-color STORM in 3-dimension. Magnified views of the indicated area in **(L)** were shown in **(M–O')**. **(P–R)** COS7 cells were co-stained with antibodies against clathrin heavy chain and AP3δ1. Localization patterns of AP-3 marked by AP3δ1 and clathrin heavy chain in the same COS7 cell were analyzed by two-color STORM in 2-dimension. Magnified views of the indicated area in panel P were shown in **(Q,R)**. **(S)** Quantification of fractions of AP-1, GGA2, AP-3, or epsinR punctae that were associated with clathrin based on super-resolution images acquired from three independent repetitions in each experimental group (mean ± SD, based on ≥9 super-resolution images). ***p < 0.001 by two-tailed Student's *t*-test. Scale bar in each panel was indicated. The super-resolution images showed the localization patterns of the indicated protein in the whole juxtanuclear area where the majority of the cargo adaptors were localized. Each dot in the graphs represents data of the whole juxtanuclear Golgi area of each individual cell.

AP-1 is a major cargo sorting complex at the TGN and it recruits clathrin to membranes during vesicle formation. Super-resolution images revealed that both AP-1, indicated by its γ1 subunit, and clathrin, marked by clathrin heavy chain (CHC), colocalized in many of the punctate structures in the juxtanuclear area (Figure 2A, and magnified view in Figures 2B,C). In addition to the AP-1 positive structures, clathrin exhibited additional locations, presumably associated with other clathrin adaptors.

We then performed 3-D super-resolution imaging analysis to analyze the spatial relationships between clathrin and AP-1 (Figures 2D–G', Videos S1–S3). 3-D super-resolution imaging analysis revealed that AP-1 can form elongated structures with over 250 nm in length (Figures 2E–F', Videos S1, S2). Quantification analysis of the super-resolution images indicates that around 46% of AP-1 structures were associated with clathrin (Figure 2S). Many of the AP-1 structures that were not associated with clathrin were small structures.

We also performed 3-D super-resolution imaging analysis of the spatial relationships between clathrin and two other cargo adaptors, epsinR and GGA2. Both epsinR and GGA2 also exhibited punctate localization patterns and many of these punctae were associated with clathrin (Figures 2H–O', Videos S4–S9). Quantification analysis indicates that around 46% of epsinR and around 44% of GGA2 structures were clathrin-associated (Figure 2S). Similar to AP-1, some epsinR and GGA2 punctae were elongated with over 250 nm in length (Figures 2I,N, Videos S4, S8). In contrast, the percentage of AP-3 structures, labeled by AP3δ1, showed a significantly reduced association with clathrin at the juxtanulear area (Figures 2P–R, and quantification in Figure 2S).

If the cargo adaptors and clathrin are assembled on vesicle membranes, they are expected to be localized on ring structures. However, we did not detect clear ring structures labeled by antibodies against cargo adaptors and clathrin in any of the super-resolution images. As a control for our analysis, we performed super-resolution imaging analysis to analyze the localization pattern of clathrin on the plasma membrane of COS7 cells. To induce endocytosis, we treated COS7 cells with epidermal growth factor (EGF) and analyzed the localization of clathrin heavy chain (CHC) 5 min after EGF treatment. We detected ring structures of CHC with the diameter around 100 nm (Figure S4A and magnified views shown in Figures S4B–D). This analysis indicates that our STORM can detect ring-localization of clathrin around the vesicle coats. A possible explanation for the failure to detect clear ring structures of clathrin at the juxtanuclear TGN area is that the area of plasma membrane that was captured in STORM was flat and attached to the coverslip in 2D, whereas the TGN has 3D shapes. Thus, it is relatively easier to capture the clathrin-coated pits (CCPs) on the plasma membrane than to capture the CCPs at the TGN. Another possibility is that the vesicle coats are unstable and are disassembled after vesicles are formed at the TGN or endosomes. It is also possible that the antigen might be inaccessible in the context of the coat or other crowded environments at the TGN or endosomes. We propose that the punctate structures detected in our super-resolution imaging analyses represent cargo adaptors and clathrin that are assembled on the microdomains on the TGN or the endosomal membranes but not on the vesicle membranes.

### Analysis of the Spatial Relationships Among the TGN/endosome-localized Cargo Adaptors

**Figure 3:**
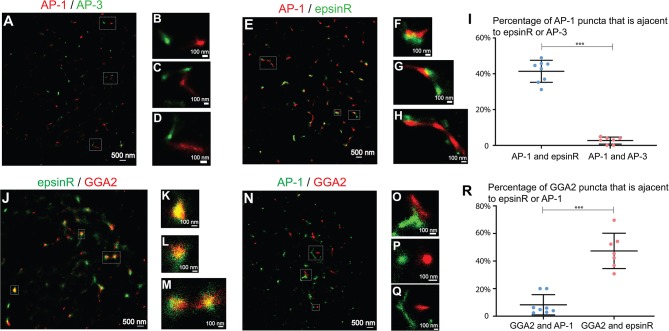
Spatial relationship analysis between clathrin adaptors at the juxtanuclear area. **(A–D,N–Q)** COS7 cells were co-stained with antibodies against γ1-adaptin and δ3-adaptin or co-stained with antibodies against γ1-adaptin and GGA2. Two-color STORM was then utilized to visualize localizations of AP-1 and AP-3 **(A–D)** and the localization of AP-1 and GGA2 **(N–Q)**. **(E–H,J–M)**. COS7 cells were transfected with epsinR-FLAG. Day 1 after transfection, cells were co-stained with antibodies against γ1-adaptin and FLAG tag **(E–H)** or with antibodies against GGA2 and FLAG tag **(J–M)**. Two-color STORM was then utilized to visualize localizations of γ1 and epsinR-FLAG and the localizations of GGA2 and epsinR-FLAG. The Magnified views of the indicated area in **(A,E,J,N)** were indicated in **(B–D,F–H,K–M,O–Q)**. The scale bar of each panel was indicated. **(I,R)** The percentage of AP-1 punctae that were associated with epsinR or AP-3 was quantified **(I)** and the percentage of GGA2 punctae that were associated with epsinR or AP-1 was quantified **(R)** (mean ± SD, based on ≥7 super-resolution images). ***p < 0.001 by two-tailed Student's *t*-test. AP-1 and AP-3 were labeled by antibodies against γ1 and δ3, respectively. The super-resolution images showed the localization patterns of the indicated protein in the whole juxtanuclear area where the majority of the cargo adaptors were localized. Each dot in the graphs represents data of the whole juxtanuclear Golgi area of each individual cell. Quantification analyses were based on super-resolution images acquired from three independent repetitions for each experimental group.

Next, we analyzed the spatial relationships among the TGN- and endosome- localized cargo adaptors at fine resolution. We first analyzed the spatial relationship between AP-1 and AP-3, which are marked by their γ1 and δ1 subunits, respectively. Super-resolution images of AP-1 and AP-3 showed that AP-1 and AP-3 remained largely separated at the juxtanuclear area (Figures 3A–D and quantification in Figure 3I) suggesting that they may mediate distinct steps in cargo sorting. We then analyzed the spatial relationship between AP-1 and epsinR. EpsinR directly interacts with the appendage domain of AP-1 (Owen et al., 2004) and colocalized with AP-1 when viewed under conventional microscope (Hirst et al., 2003). Super-resolution imaging analysis of AP-1 and epsinR showed that some of the epsinR structures were associated with AP-1 structures (Figures 3E–H, and quantification in Figure 3I) and some of the AP-1 and epsinR structures were separated from one another (Figures 3E,G). EpsinR also interacts with the GAE domain of GGA2. Similar to the spatial relationship between AP-1 and epsinR, we found that some GGA2 punctae were associated with epsinR punctae (Figures 3J–M). Quantification analysis indicates that around 41% of the AP-1 structures and 47% of the GGA2 structures were spatially associated with epsinR (Figures 3I,R).

We then analyzed the spatial relationships between AP-1 and GGA2. In yeast, these two cargo adaptors are adjacent to, but distinct from one another, and Gga proteins and AP-1 are sequentially assembled at the TGN (Daboussi et al., 2012). In yeast, a key GGA protein, Gga2p, directly binds phosphatidylinositol (PtdIns) 4-kinase (PI4K), Pik1 (Daboussi et al., 2017), which recruit Pik1 to the TGN to produce PtdIns4P and induce a second wave of AP-1 assembly (Daboussi et al., 2012, 2017). The sequential assembly process allows assembly of GGA proteins and AP-1 to be separated in time and space. Mammalian GGA2 also directly interacts with the Pik1 homolog, PI4KIIIβ, and regulates its membrane association (Daboussi et al., 2017). A poor colocalization between GGA1 and AP-1 was detected in mammalian cells (Mardones et al., 2007). Here, we found that around 8% of the GGA2 punctae were spatially associated with the AP-1 punctae and the majority of AP-1 and GGA2 punctae were separated in COS7 cells (Figures 3N–Q and quantification in Figure 3R).

### Visualization of Sorting of Planar Cell Polarity Proteins, Vangl2 and Frizzled6, Upon TGN Exit Through Super-Resolution Imaging Analysis

**Figure 4:**
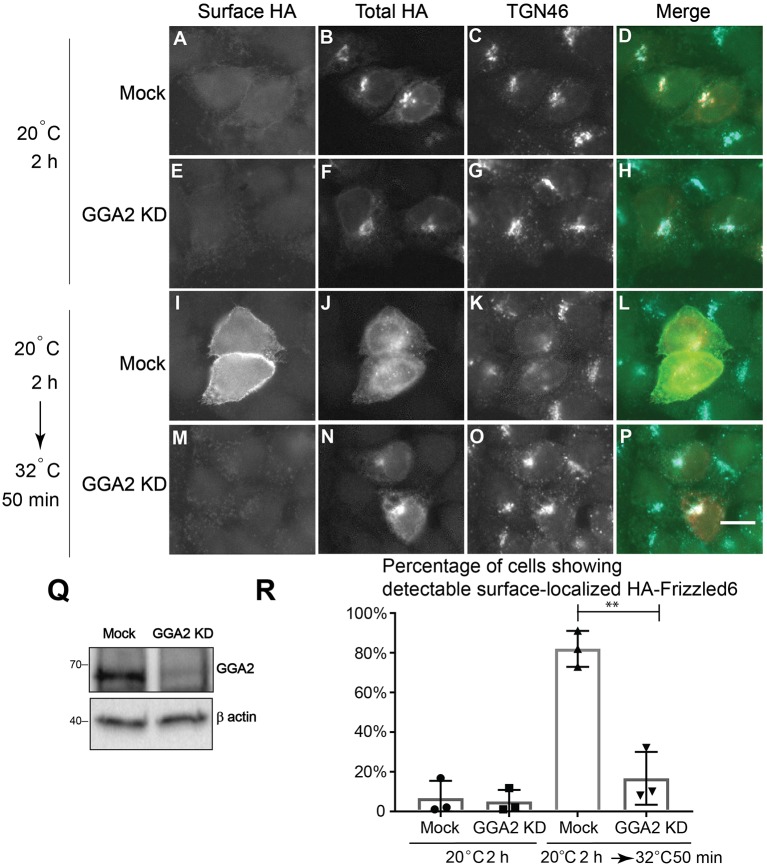
GGA2 regulates TGN export of Frizzled6. **(A–P)** HeLa cells were mock transfected **(A–D,I–L)** or transfected with siRNA against GGA2 **(E–H,M–P)** and re-transfected after 48 h with plasmids encoding HA-Frizzled6 **(A–P)**. On day 3 after knockdown, cells were incubated at 20°C for 2 h **(A–H)** or incubated at 20°C for 2 h then shifted to 32°C for 50 min **(I–P)** in the presence of cycloheximide. After incubation, cells were analyzed by immunofluorescence. The surface-localized HA-Frizzled6 and the total HA-Frizzled6 were stained by mouse and rabbit anti-HA antibodies, respectively. Scale bar, 10 μm. **(Q)** HeLa cells were mock transfected or transfected with siRNA against GGA2. On day 3 after transfection, cells were analyzed by immunoblot. **(R)** Quantification of the percentage of cells showing detectable surface localized Frizzled6 in cells treated with control siRNA or siRNA against GGA2 after incubation at 32°C (mean ± SD; *N* = 3; >100 cells counted for each experiment). **p < 0.01 by two-tailed Student's *t*-test.

Our analyses indicate that GGA2 and AP-1 are largely spatially segregated from each other suggesting that GGA2 and AP-1 can function to mediate sorting of distinct cargo proteins at the TGN and endosomes. AP-1 has been shown to regulate sorting of a planar cell polarity protein, Vangl2, at the TGN (Guo et al., 2013). TGN sorting of another PCP protein, Frizzle6, which is localized on the cellular boundaries opposing to Vangl2, is independent of AP-1 but dependent on epsinR (Ma et al., 2018). Our super-resolution imaging analyses indicate that epsinR showed an AP-1-associated pattern and a GGA2-associated pattern. This observation prompted us to test whether it is the GGA2-associated epsinR that regulates sorting of Frizzled6 at the TGN. To test this, we first analyzed whether GGA2 is important for TGN export of Frizzled6. Cells treated with siRNA against GGA2 did not show detectable defects in HA-Frizzled6 localization at the steady state, possibly due to the presence of functional redundancy. To test whether knockdown of GGA2 causes a delay in the kinetics of TGN export of Frizzled6, we incubated HeLa cells at 20°C in the presence of cycloheximide to accumulate newly-synthesized HA-Frizzled6 in the TGN. As the HA tag is exposed at the extracellular domain of Frizzled6, we performed a surface labeling experiment to detect the surface-localized HA-Frizzled6. After a 20°C incubation, HA-Frizzled6 had accumulated at the juxtanuclear area, colocalized with TGN46 and the majority of cells showed no detectable surface-localized Frizzled6 (Figures 4A–H, and quantification in Figure 4R). When cells were shifted to 32°C, Frizzled6 in most control siRNA treated cells showed a detectable surface-localized pattern (Figures 4I–L, and quantification in Figure 4R). Through this method, we found that cells treated with siRNA against GGA2, which efficiently reduced the expression of GGA2 (Figure 4Q), showed a significant reduction of the percentage of cells showing surface-localized Frizzled6 (Figures 4M–P, and quantification in Figure 4R). In contrast, knockdown of GGA2 did not cause a defect in the TGN export of HA-Vangl2, whereas the majority of cells treated with siRNA against CHC showed strong accumulations of HA-Vangl2 at the juxtanuclear area (Figure S5). Using a similar temperature shift approach, we detected a significant reduction of the percentage of cells showing surface-localized Frizzled6 in GGA3 knockdown cells but not GGA1 (Figure S6), indicating that GGA3 and GGA2 may redundantly regulate sorting of Frizzled6 at the TGN.

**Figure 5:**
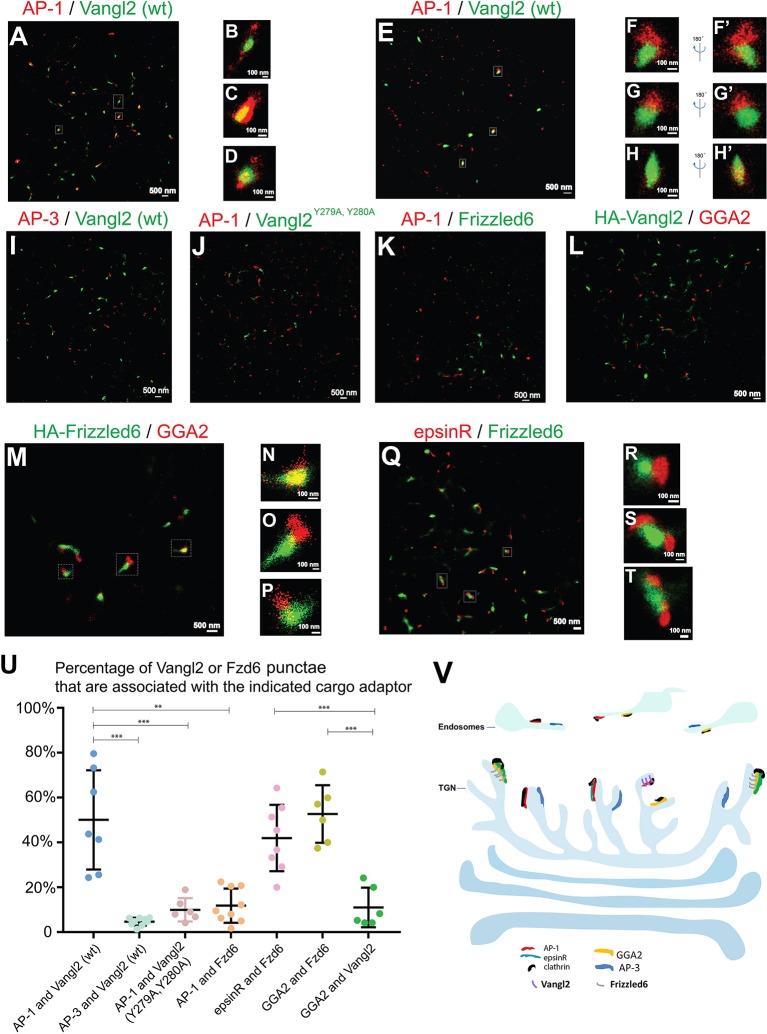
Visualization of sorting of Vangl2 and Frizzled6 upon exiting the TGN through two-color STORM. **(A–H')** COS7 cells were transfected with HA-Vangl2 (wt). Day 1 after transfection, cells were incubated at 20°C for 2 h and then at 32°C for 5 min. After incubation, two-color STORM was utilized to visualize localizations of Vangl2 (wt) and the γ subunit of AP-1 at 2-dimension **(A–D)** and 3-dimension **(E–H')**. Magnified views of the indicated areas in **(A)** were shown in **(B–D)**. Magnified views of the indicated areas in **(E)** were shown in **(F–H)**. 180° rotated views of the **(F–H)** were shown in **(F'–H')**. The scale bar of each image was indicated. **(I–K)** COS7 cells were transfected with HA-Vangl2 (wt) or HA-Vangl2 ^Y279A, Y280A^ or HA-Frizzled6. Day 1 after transfection, cells were incubated at 20°C for 2 h and then at 32°C for 5 min. After incubation, two-color STORM was utilized to visualize localizations of Vangl2 (wt) and the δ subunit of AP-3 **(I)**, Vangl2 ^Y279A, Y280A^ and the γ subunit of AP-1 **(J)**, Frizzled6 and the γ subunit of AP-1 **(K)**. Scale bar, 500 nm. **(L–Q)** COS7 cells were transfected with HA-Vangl2 **(L)** or HA-Frizzled6 **(M–P)** or co-transfected with epsinR-FLAG and HA-Frizzled6 **(Q–T)**. Day 1 after transfection, cells were incubated at 20°C for 2 h and then at 32°C for 5 min. After incubation, two-color STORM was utilized to visualize localizations of Vangl2 and GGA2 **(L)**, Frizzled6 and GGA2 **(M–P)** or epsinR-FLAG and Frizzled6 **(Q)**. Magnified views of the indicated areas in **(M,Q)** are shown in **(N–P,R–T)**. **(U)** Quantifications of the percentage Vangl2 or Frizzled6 punctae that are associated with the indicated cargo adaptor (mean ± SD, based on ≥6 super-resolution images in each experimental group). **p < 0.01, ***p < 0.001 by two-tailed Student's *t*-test. The super-resolution images showed the localization patterns of the indicated protein in the whole juxtanuclear area where the majority of the cargo adaptors were localized. Each dot in the graphs represents data of the whole juxtanuclear Golgi area of each individual cell. Quantification analyses were based on super-resolution images acquired from three independent repetitions for each experimental group. **(V)** The proposed model showing that AP-1, AP-3, epsinR, and GGA2 are assembled into elongated structures on distinct microdomains on TGN and endosomal membranes with distinct spatial relationships: the spatial segregated GGA2 and AP-1 regulates sorting of Frizzled6 and Vangl2, respectively; the spatially associated cargo adaptor, GGA2 and epsinR, cooperatively regulate sorting of Frizzled6.

Next we sought to visualize these sorting events using two-color STORM. We first investigated whether Vangl2 was enriched in membrane patches coated by AP-1 upon TGN exit. COS7 cells expressing HA-Vangl2 were incubated at 20°C to accumulate newly-synthesized Vangl2 at the TGN. We analyzed the spatial relationship between Vangl2 and AP-1 after the cells were incubated at 32°C for 5 min to restore the protein sorting process. In this incubation, the extent of exit from the TGN was minimal and the majority of cargo proteins remained localized at or around the juxtanuclear area (Figure S7). We selected the juxtanuclear area where the majority of Vangl2 was localized for the super-resolution imaging analysis. STORM images of Vangl2 and AP-1 in 2- and in 3-dimensions showed that many Vangl2 structures are associated with AP-1 (Figure 5A and the magnified view in Figures 5B–E and the magnified view in Figures 5F–H', Videos S10–S12). A broad range in the degree of co-localization was apparent from a quantification of 7 super-resolution images from 7 cells (Figure 5U), possibly due to an asynchronous budding of vesicles from the TGN followed by coat protein release from the membrane. As a control, we analyzed the spatial relationship between Vangl2 and AP-3. The majority of Vangl2 structures were not associated with the AP-3 (Figures 5I,U). As another control, we analyzed the spatial relationship of AP-1 and Vangl2 bearing mutations in the tyrosine sorting motif (Vangl2 ^Y279A, Y280A^), which we previously implicated in the interaction with AP-1 (Guo et al., 2013). STORM imaging analysis showed that the majority of Vangl2 ^Y279A, Y280A^ punctate structures were not adjacent to AP-1 (Figures 5J,U).

To test whether Frizzled6 is associated with AP-1 upon TGN exit, we analyzed the spatial relationship between HA-tagged Frizzled6 and AP-1 under the same conditions of temperature shift. Super-resolution imaging analysis indicated that the majority of Frizzled6 punctae were not associated with AP-1 (Figures 5K,U) consistent with our previous report that sorting of Frizzled6 is not mediated by AP-1. Moreover, we found that many Frizzled6 structures but not Vangl2 structures were associated with GGA2 under the temperature shift conditions (Figures 5L–P). Quantification of 6 super-resolution images from 6 cells of each group indicate that the percentage of Frizzled6 punctae that were associated with GGA2 is significantly higher than the percentage of Vangl2 punctae that were associated with GGA2 (Figure 5U). These analyses indicate that GGA2 regulates TGN export of Frizzled6 but not Vangl2 and provide direct evidence demonstrating sorting of specific cargo proteins by specific cargo adaptors at the TGN.

We recently showed that TGN export of Frizzled6 is also regulated by epsinR. We found that many Frizzled6 structures were associated with epsinR under the temperature shift conditions (Figures 5Q–T). Quantification analysis indicates that the percentage of Frizzled6 punctae that were associated with epsinR is significantly higher than the percentage of Frizzled6 punctae that were associated with AP-1 (Figure 5U). These analyses indicate that sorting of Frizzled6 at the TGN is regulated by the GGA2-associated epsinR but not the AP-1-associated epsinR.

Next, we analyzed the spatial relationships between Vangl2 and Frizzled6 upon TGN exit in COS7 cells co-transfected with HA-Frizzled6 and Myc-Vangl2. After an incubation at 20°C, we observed that many Frizzled6 and Vangl2 structures overlapped at the juxtanuclear area (Figures S8A–S8C), suggesting that they were intermixed with each other on the TGN membranes in a condition of blocked exit from the TGN. After the temperature was released at 32°C for 5 min, we found the majority of Vangl2 and Frizzled6 were not overlapped (Figures S8D–S8F, and quantification in Figure S8G), suggesting that Vangl2 and Frizzled6 spatially segregate on exit from the TGN.

## Discussion

Protein sorting at the TGN and endosomes play important roles in targeting various cargo proteins to their specific destinations. A number of cargo adaptors have been identified to mediate this essential cellular process but their spatial relationships at high resolution have not been systematically analyzed. Using two-color STORM imaging, we demonstrated that the TGN- and endosome-localized cargo adaptors, AP-1, AP-3, GGA2, and epsinR, are enriched in distinct microdomains (Figure 5V). These cargo adaptors can form elongated structures of over 250 nm in length. Many of the elongated structures formed by GGA2, AP-1, and epsinR are associated with clathrin. In contrast, AP-3 showed a significantly reduced association with clathrin compared to the other three cargo adaptors. In addition, we showed that the majority of AP-3 structures are segregated from AP-1 structures (Figure 5V). Similar observations of these spatial relationship have been detected on the buds arising from endosome-associated tubules by immunogold electron microscopy (Peden et al., 2004).

Our high resolution imaging analysis also revealed that AP-1 structures are largely segregated from GGA2 structures and there are at least two populations of epsinR, one associated with AP-1 and the other associated with GGA2 (Figure 5V). In yeast, Ent3 and Ent5, the yeast homologs of epsinR, were shown by structured illumination microscopy (SIM) to be predominantly colocalized with Gga2p and AP-1, respectively. Gga2p and AP-1 are adjacent to but distinct from each other (Daboussi et al., 2012). These observations suggest that the spatial relationships among the cargo adaptors are conserved from yeast to mammalian cells.

The resolution of a STORM image can be indicated by the mean localization error of single molecular fluorescence. We examined the STORM resolution by analyzing the localization error distribution of single molecular fluorescence from the localization table of the raw STORM data of Figure 5A (Figure S9) and find the mean localization error of 11.2 nm. The resolution of STORM images is also affected by low localization density, but we do not have any structure that is too sparse to affect the conclusions. Besides, we have a robust protocol for labeling and tested different structures and no such issues were found (Huang et al., 2017; Liu et al., 2017; Cheng et al., 2018; Mao et al., 2019). Moreover, we measured the density of localization in Figures 3A,E, 5A,I to be >18 counts/nm^2^ at the least dense region, which means the average distance between adjacent localized sites is below 20 nm. These evidence suggests that our STORM system can achieve 20 nm resolution.

Both epsinR and GGA2 regulate TGN export of Frizzled6 but not Vangl2, suggesting that these two spatially associated cargo adaptors can cooperatively regulate a specific protein sorting process. This co-operativity has also been detected in yeast. Ent3p, which spatially associates with Gga2p, functions primarily in GGA-dependent transport (Costaguta et al., 2006). Ent3p, but not Ent5p, facilitates binding of Gga2p to the endosomal syntaxin Pep12p (Copic et al., 2007). In contrast, Ent5p, which spatially associates with AP-1, is more critical for AP-1-mediated transport (Costaguta et al., 2006).

EpsinR contains an N-terminal ENTH domain and a C-terminal unfolded region. The C-terminal unfolded region of epsinR directly interacts with the appendage domain of AP-1 (Owen et al., 2004) indicating that this direct interaction may induce their spatial association. Interestingly, we recently found that Frizzled6 also interacts with the C-terminal unfolded region of epsinR (Ma et al., 2018). The interaction between Frizzled6 and epsinR causes dissociation of epsinR from AP-1 (Ma et al., 2018). This result indicates that the presence of Frizzled6 can induce the separation of epsinR from AP-1 to allow these two cargo adaptors to perform distinct cargo sorting functions (Ma et al., 2018). In mammalian cells, knockdown of either AP-1 or epsinR causes reduction of the other in clathrin coated vesicles (Hirst et al., 2004). Acute inactivation of epsinR blocked the production of the entire population of clathrin-coated vesicles, suggesting a more global function of epsinR (Hirst et al., 2015). The functional role of epsinR remains to be elucidated in the AP-1-mediated vesicular trafficking process.

Spatially segregated assembly of cargo adaptors, such as GGA2 and AP-1, may provide a mechanism to allow these cargo adaptors to package distinct cargo molecules into transport vesicles. We previously showed that TGN export of Vangl2 but not Frizzled6 depends on AP-1 (Ma et al., 2018). Here, we have shown that GGA2 regulates TGN export of Frizzled6 but not Vangl2 suggesting that sorting of Vangl2 and Frizzled6 is regulated by the spatially segregated cargo adaptors, AP-1 and GGA2, respectively. Moreover, through super-resolution imaging analysis, we observed specific associations of Vangl2 and Frizzled6 with AP-1 and GGA2, respectively, upon exiting the TGN, confirming this differential sorting process.

How can cargo adaptors be recruited onto spatially segregated microdomains on the TGN or endosomes? One possible explanation is that the spatial separation is caused by a sequential assembly process as exemplified by GGAs and AP-1. In this scenario, assembly of GGAs on TGN membranes recruits PI4 kinase which in turn generates PI4P that induces a second wave of AP-1 assembly (Daboussi et al., 2012, 2017). Thus a peak of GGA assembly at a specific site will be followed by a peak of AP-1 assembly (Daboussi et al., 2012). Such a sequential assembly process correlates with sequential cargo sorting events (McDonold and Fromme, 2014). This process may prevent the assembly of GGA and AP-1 at the same time and space. Another possible explanation is that binding of cargo adaptors to specific cargo molecules may cause polymerization of the cargo adaptors. This polymerization process will enrich the cargo adaptors as well as their associated cargo molecules into specific membrane domains. This hypothesis is supported by evidence showing that binding of cargo to AP-1 promotes polymerization of AP-1 (Meyer et al., 2005; Lee et al., 2008). In addition to inducing polymerization of cargo adaptors, cargo molecules can stabilize the membrane association of cargo adaptors to allow sufficient time for vesicle coat assembly (Crottet et al., 2002; Lee et al., 2008; Caster et al., 2013; Guo et al., 2013, 2014; Ren et al., 2013). Thus, cargo molecule and cargo adaptors can form a positive feedback loop which will induce assembly of specific cargo sorting machinery on distinct membrane microdomains.

Cargo adaptors that mediate protein sorting at the TGN and endosome are present in a sophisticated interaction network and defects in these cargo adaptors can directly or indirectly influence the functions of other cargo adaptors. Even the spatially segregated cargo adaptors are not functionally independent of each other. Evidence suggests that acute inactivation of AP-1 in mammalian cells using the “knocksideways” system depletes GGA2 from clathrin coated vesicles and causes stronger defects in cargo sorting than acute inactivation of GGA2 does (Hirst et al., 2012). However, knockdown analysis indicates that incorporation of GGA2 into clathrin coated vesicles is not affected by AP-1 depletion and vice versa (Hirst et al., 2009), suggesting that knock down cargo adaptors produces a weaker phenotype than knocking the cargo adaptors sideways. Deletion AP-1 but not GGA2 by siRNA knockdown regulates HIV protein Nef-mediated downregulation of histocompatibility complex (MHC) class I (Hirst et al., 2009), which is consistent with our study indicating that these two cargo adaptors can function to regulate sorting of distinct cargo molecules.

We used COS7 cells for the spatial analysis. The spatial relationships of the cargo adaptors are consistent with the spatial relationships of cargo adaptors observed in yeast and human fibroblasts (Hirst et al., 2003; Mardones et al., 2007). An important future step of our study is to measure the extent of co-localization using polarized cells or when cells are in different differentiation states. Because of the limitation of two color, we cannot distinguish whether the signal we detected was located at the Golgi or endosomes. Another important future step of our study is to build a three-color super-resolution imaging system to demonstrate where the segregation took place. As the majority of the signal of AP-1, GGA2, and epsinR were located at the juxtanuclear Golgi area and the super-resolution images showed the localization patterns of these cargo adaptors in the whole juxtanuclear Golgi area, we propose that the majority the signal detected in this study was from the Golgi.

Altogether, our high-resolution imaging analysis indicates that cargo adaptors, AP-1, AP-3, and GGA2, are assembled into large elongated structures on distinct exit domains at the TGN or endosomes to mediate sorting of specific cargo molecules (Figure 5V). The spatially segregated GGA2 and AP-1 regulates sorting of Frizzled6 and Vangl2, respectively. And the spatially associated cargo adaptor, GGA2 and epsinR, cooperatively regulate sorting of Frizzled6.

## Materials and Methods

### Cell Lines, Antibodies, and Plasmids

HeLa cells were kindly provided by the University of California-Berkeley Cell Culture Facility and were confirmed by short tandem repeat profiling. COS-7 cells were obtained from ATCC (Cat#ATCC CRL-1651, RRID: CVCL_0224). The cells were tested negative for mycoplasma contamination. COS7 and HeLa cells were maintained in GIBCO Dulbecco's Modified Eagle Medium containing 10% Fetal Bovine Serum (FBS), 10 mU/ml of penicillin and 0.1 mg/ml of streptomycin. Transfection of DNA constructs into COS7 cells and immunofluorescence were performed as described (Guo et al., 2013). Temperature shift experiment was performed as described (Guo et al., 2013).

The commercial antibodies were: mouse anti-golgin-97 (Invitrogen #A21270, RRID: AB_221447), mouse anti-GM130 (BD Biosciences #610823, RRID: AB_398142), mouse anti-γ1-adaptin (BD Bio #610385, RRID: AB_397768), rabbit anti-HA (Cell Signaling #3724, RRID: AB_1549585), rabbit anti-clathrin heavy chain (abcam #ab21679, RRID: AB_2083165), mouse anti-δ subunit of AP-3 (Developmental Studies Hybridoma Bank, number anti-delta SA4, RRID: AB_2056641), sheep anti-TGN46 (AbD Serotec, number AHP500G, RRID: AB_323104), mouse anti-FLAG (Sigma, number F3165, RRID: AB_259529), rabbit anti-FLAG (Sigma, number F7425, RRID: AB_439687), rabbit anti-γ1-adaptin (Proteintech, number 13258-1-AP, RRID: AB_2058209), mouse anti-GGA2 (BD Bioscience, number 612612, RRID: AB_399892), mouse anti-HA (BioLegend, number 901501, RRID: AB_2565006), rabbit anti-GGA1 (Thermo Fisher Scientific, number PA5-12130, RRID: AB_2232367), rabbit anti-GGA3 antibody (Cell Signaling Technology, number 4167s, RRID: AB_1903987).

The plasmid encoding HA-tagged mouse Vangl2 was cloned in pCS2 (Merte et al., 2010). The plasmid encoding HA-Frizzled6 was generated by cloning full length mouse Frizzled6 amplified from Frizzled6-Myc into pcDNA4/TO and an HA tag was inserted by PCR between F22 and T23 (Ma et al., 2018). The plasmid encoding FLAG-tagged full length epsinR was generated by cloning full length epsinR from human epsinR cDNA (OriGene) into p3XFLAG-CMV-14 (Sigma Aldrich).

The target sequence of the siRNAs against clathrin heavy chain, AP1γ1-adaptin and AP3δ1-adaptin were described previously (Ma et al., 2018). The target sequence of siRNA against GGA2 was: CCGGAAGACATCAAGATTCGA. The target sequence of siRNA against GGA1 was GCCGAAGAATGTGATCTTT, the target sequence against GGA3 was GTGAGATGCTGCTTCATTA.

### Transfection and Immunofluorescence Staining

Transfection of siRNA or DNA constructs into HeLa cells or COS7 cells and immunofluorescence were performed as described (Guo et al., 2013). Cells growing on coverslips were fixed in 4% PFA for 20 min then washed with PBS and incubated with permeabilization buffer (2.5% FBS, 0.1% TX-100, 0.2 M Glycine in PBS) at RT for 30 min. Then cells were sequentially incubated with primary antibody and secondary antibody diluted in permeabilization buffer for 30 min. Each antibody incubation was following by five times wash with PBS. After staining, cells were fixed again in 4% PFA for 20 min and then washed with PBS. Surface labeling procedure was described (Ma et al., 2018). Briefly, to label surface-localized Frizzled6 containing an HA tag in its extracellular domain, HeLa cells expressing HA-Frizzled6 were incubated with the mouse anti-HA antibody (1:200) in PBS containing FBS (250 μl in 10 ml) for 40 min on ice. After several washes with PBS, cells were fixed for 15 min with 4% paraformaldehyde in PBS and then a normal immunofluorescence procedure was performed. Fluorescent images were acquired with a Zeiss Axioobserver Z1 microscope system.

### Quantitative Real-Time PCR

HeLa cells transfected with control siRNA or AP3δ1 siRNA. Seventy two hours after transfection, HeLa cells were harvested for RNA extraction. Total RNA was extracted using TRIzol reagent (Invitrogen, Catalog number: 15596026). Subsequently, RT-qPCR were performed as described previously (Amatori et al., 2017). RT-qPCR experiment was conducted in technical triplicates. 2-ΔΔCT method was used to detect the gene expression variation between the control group and the knockdown group.

### Super-Resolution Imaging Analysis

The two-color STORM was built as described (Wang et al., 2016). Briefly, this STORM was built using a Nikon Ti-E inverted microscope with perfect focus system. A 656.5 nm DPSS laser and a 750 nm diode laser were used to provide laser excitations for Alexa 647 channel and Alexa 750 channel, respectively. A 100 × TIRF objective (Nikon) was used to collect the fluorescent signals of both channels. Subsequently, the fluorescent signals passed through a home-built channel splitter before simultaneously forming images on an EMCCD (Andor, IXon-Ultra). Emission filters (Chroma ET700/50 m and ET810/90 m) were placed in each path of the channel to block the excitation laser.

The imaging buffer for two-color STORM was designed for the two dye combination Alexa 647 and Alexa 750 (Zhao et al., 2015). The buffer contained 10% (w/v) glucose, 25 mM Tris(2-carboxyethyl)phosphine hydrochloride solution (TCEP, Sigma-Aldrich 646547), 2 mM cyclooctatetraene (Sigma-Aldrich 138924), 560 μg/ml glucose oxidase, 40 μg/ml catalase, 50 mM Tris-Cl pH 8.0, 1 mM ascorbic acid and 1 mM methyl viologen. The composition of the imaging buffer provided matched and balanced switching characteristics for both dyes (Zhao et al., 2015). The sample was mounted on a customized glass-bottom chamber filled with imaging buffer. The desired position is identified using conventional fluorescence image with relatively low laser excitation power, typically 60 W/cm^2^ for a 656.5 nm laser and 80 W/cm^2^ for a 750 nm laser. After the region of interest was identified, the laser power was increased to 4 kW/cm^2^ in both channels enabling rapid “blinking” of dye molecules for single molecule detection and localization. The blinking was recorded by an EMCCD with 100 × EM gain at 30 Hz for 15,000 to 20,000 frames based on the abundance of proteins. When each frame was captured, the peak finding algorithm recognized the sites of blinking, followed by the fitting algorithm that determined the centroid of each blinking with nanometer accuracy. These centroids were registered to the final super-resolution image. In addition, active sample locking was applied to stabilize the sample with nanometer accuracy in the x-y plane and z-axis during acquisition. Optical astigmatism was used to achieve 3-dimensional STORM (Huang et al., 2008). The data acquisition and single-molecule localization are carried out with software called Rohdea developed by NanoBioImaging Ltd, while the rendering is done with QuickPALM (Henriques et al., 2010) from Fiji (win64 version, National Institutes of Health, USA). Gaussian rendering is used to simulate sub-diffraction spot. Fiji plug-in 3D viewer was used to record 3D rotation films.

Fiji was used for calculating the percentage of overlapped pixels based on a modified quantification method (Guo et al., 2008), utilizing the following procedures: (1) a threshold equaling to 25% of the maximal pixel value of each marker was chosen; (2) the average pixel intensity for the two thresholded images was equalized using the divide function; (3) the number of above-threshold pixels for each marker was determined per cell; (4) the number of above-threshold overlapped pixels was determined using the colocalization highlight function in MBF plugin collection (ImageJ) with a fixed ratio of 0.6; (5) for each marker, the number of overlapped pixels was divided by the number of above-threshold pixels to yield the percentage of a given maker's area in a cell that overlapped with the other marker; (6) the average of the two values, each representing the percentage of overlapped pixels for each marker, was used as final value indicating the percentage of overlapped pixels.

To measure the percentage of the punctate structures in one channel (channel A) that were associated with the punctate structures in the other channel (channel B), the number of punctates in channel A that were associated with the punctate structures in channel B was divided by the total number of the punctates in channel A in each super-resolution image. The number of punctate structures in each super-resolution image was counted manually.

## Data Availability

The datasets generated for this study are available on request to the corresponding author.

## Author Contributions

YH, TM, PL, JW, TZ, and YG: data curation, formal analysis, validation, and investigation. SD, ML, and YG: resources and supervision. YG: conceptualization, writing-review and editing, writing original draft, and project administration.

### Conflict of Interest Statement

The authors declare that the research was conducted in the absence of any commercial or financial relationships that could be construed as a potential conflict of interest.

## Supplementary Material

Figure S1 Localization patterns of imaging beads coated with both Alexa 750 and Alexa 647 revealed through two-color STORM. **(A)** Alexa 750 and Alexa 647 showed a perfectly overlapped pattern. Scale bar, 500 nm. **(B–D)** Magnified views of the area indicated by arrows in **(A)**. Scale Bar, 50 nm.

Figure S2 The localization patterns of clathrin and cargo adaptors in COS7 cells. COS7 cells were untransfected **(A–O)** or transfected with plasmids encoding FLAG-tagged epsinR **(P–R)**. Day 1 after transfection, cells were analyzed by immunofluorescence utilizing the indicated antibodies. Representative fluorescent images were shown. Scale bar, 10 μm.

Figure S3 The localization patterns of clathrin and the indicated cargo adaptors in HeLa cells. HeLa cells were transfected with control siRNA **(A,C,E,G,I)** or transfected with siRNA against the indicated cargo adaptors **(B,D,F,H)** or clathrin heavy chain **(J)**. Day 3 after transfection, cells were analyzed by immunofluorescence utilizing the indicated antibodies. Representative fluorescent images were shown. Scale bar, 10 μm. **(K–M)** HeLa cells were mock transfected or transfected with siRNA against AP1γ1, clathrin heavy chain or AP3δ1. On day 3 after transfection, cells were analyzed by immunoblot **(K,L)** or RT-PCR **(M)**.

Figure S4 The localization patterns of clathrin heavy chain on the plasma membrane of COS7 cells after stimulating with epidermal growth factor (EGF). **(A)** COS7 cells were treated with 5 ng/ml EGF for 5 min and then stained with antibodies against clathrin heavy chain. Localization patterns of clathrin heavy chain were analyzed by STORM. Scale Bar, 500 nm. **(B–D)** Magnified views of the structures highlighted by arrows in **(A)**. Scale Bar, 100 nm.

Figure S5 TGN export of Vangl2 is independent of GGA2. **(A–I)** HeLa cells were mock transfected **(A–C)** or transfected with siRNA against clathrin heavy chain (CHC, **D–F**) or transfected with siRNA against GGA2 **(G–I)** and re-transfected after 48 h with plasmids encoding HA-Vangl2 **(A–I)**. On day 3 after knockdown, cells were incubated at 20°C for 2 h then shifted to 32°C for 50 min in the presence of cycloheximide. After incubation, cells were analyzed by immunofluorescence. Scale bar, 10 μm. **(J)** Quantification of the percentage of cells showing TGN-accumulated Vangl2 in cells treated with control siRNA or siRNA against CHC or GGA2 after incubation at 32°C (mean ± SD; *N* = 3; >150 cells counted for each experiment).

Figure S6 Knockdown of GGA3 but not GGA1 causes defects in surface delivery of Frizzled6. (**A–L)** HeLa cells were mock transfected **(A–D)** or transfected with siRNA against GGA1 **(E–H)** or siRNA against GGA3 **(I–L)** and re-transfected after 48 h with plasmids encoding HA-Frizzled6. On day 3 after knockdown, cells were incubated at 20°C for 2 h then shifted to 32°C for 50 min in the presence of cycloheximide. After incubation, cells were analyzed by immunofluorescence. The surface-localized HA-Frizzled6 and the total HA-Frizzled6 were stained by mouse and rabbit anti-HA antibodies, respectively. Scale bar, 10 μm. **(M)** HeLa cells were mock transfected or transfected with siRNA against GGA1 or GGA3. On day 3 after transfection, cells were analyzed by immunoblot. **(N)** Quantification of the percentage of cells showing detectable surface localized Frizzled6 in cells treated with control siRNA or siRNA against GGA1 or GGA3 after incubation at 32°C (mean ± SD; *N* = 3; >100 cells counted for each experiment). ***p* < 0.01 by two-tailed Student's *t*-test.

Figure S7 Localizations of Vangl2 and Frizzled6 after temperature shit. **(A–F)** COS7 cells were transfected with HA-Vangl2 **(A–C)** or HA-Frizzled6 **(D–F)**. Day 1 after transfection, cells were incubated at 20°C for 2 h and then at 32°C for 5 min. The localizations of Vangl2, Frizzled6, and TGN46 was then analyzed by immunofluorescence. Scale bar, 10 μm.

Figure S8 Analysis of the spatial relationships between Vangl2 and Frizzled6 upon exiting the TGN. **(A–F)** COS7 cells were co-transfected with HA-Frizzled6 and Myc-Vangl2. Day 1 after transfection, cells were incubated at 20°C for 2 h **(A–C)** or incubated at 20°C for 2 h and then at 32°C for 5 min **(D–F)**. After incubation, cells were stained with antibodies against HA tag and Myc tag. Two-color STORM was then utilized to visualize localizations of Vangl2 and Frizzled6. **(G)** Quantification of the percentage of overlapped pixels between Vangl2 and Frizzled6 in cells incubated at 20°C for 2 h or in cells incubated at 20°C for 2 h and then at 32°C for 5 min (mean ± SD, based on three super-resolution images each). ****p* < 0.001 by two-tailed Student's *t*-test.

Figure S9 The histogram of the localization error of Figure 5A. The software rejects any fitting with error >20 nm.

Videos S1–S3 360° rotated views of AP-1 (red) and clathrin (green) of the indicated areas in Figure 2D.

Videos S4–S6 360° rotated views of epsinR (red) and clathrin (green) of the indicated areas in Figure 2H.

Videos S7–S9 360° rotated views of GGA2 (red) and clathrin (green) of the indicated areas in Figure 2L.

Videos S10–S12 360° rotated views of AP-1 (red) and Vangl2 (green) of the indicated areas in Figure 5E.
