# Supplementary figures and images for: Visualization of Protein Sorting at the Trans-Golgi Network and Endosomes Through Super-Resolution Imaging
Source: Front Cell Dev Biol. 2019 Sep 3;7:181. doi: 10.3389/fcell.2019.00181 (PMC6733968; doi:10.3389/fcell.2019.00181)

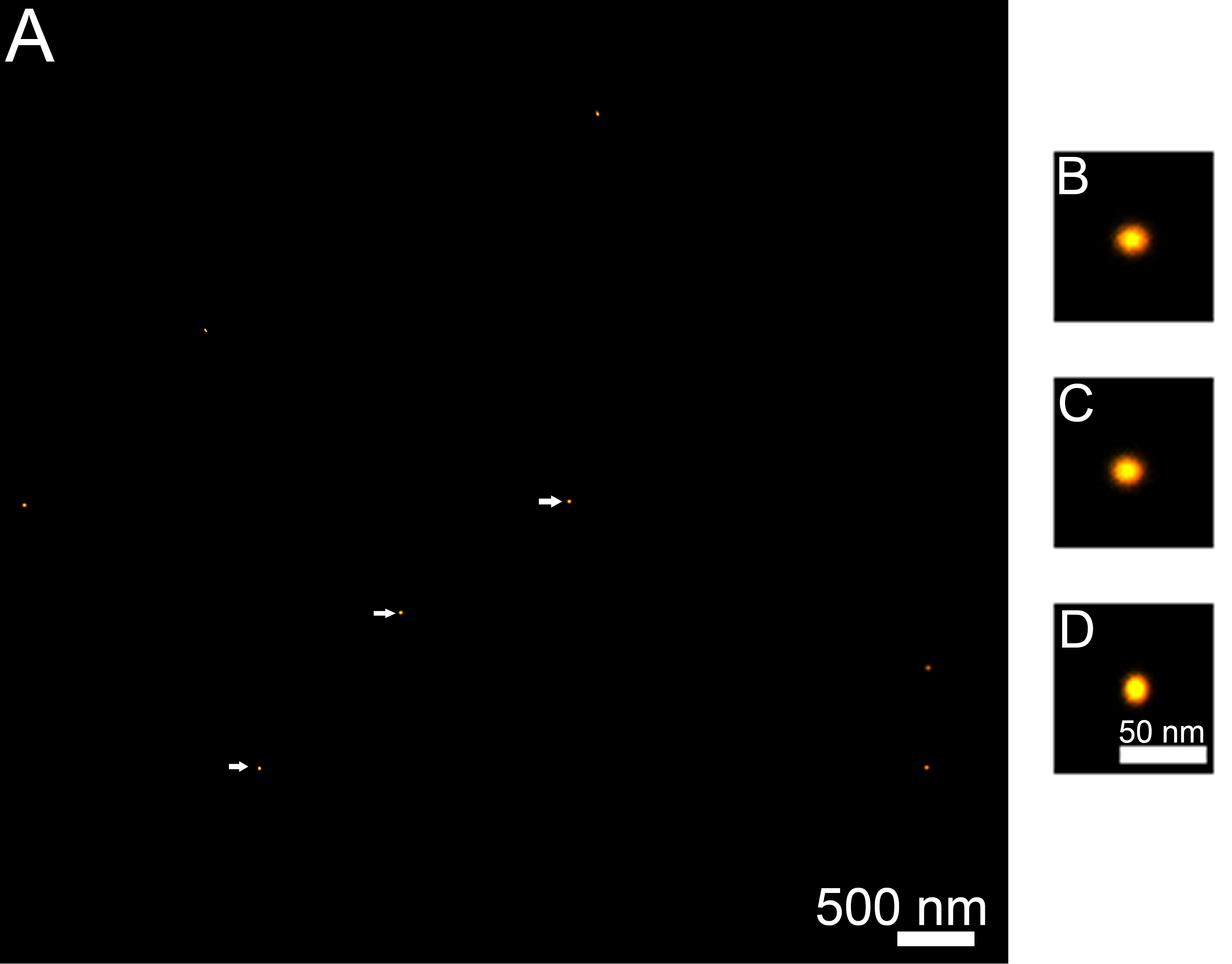

Supplement: Figure S1 — Localization patterns of imaging beads coated with both Alexa 750 and Alexa 647 revealed through two-color STORM. (A) Alexa 750 and Alexa 647 showed a perfectly overlapped pattern. Scale bar, 500 nm. (B–D) Magnified views of the area indicated by arrows in (A). Scale Bar, 50 nm. [file Image_1.jpg]

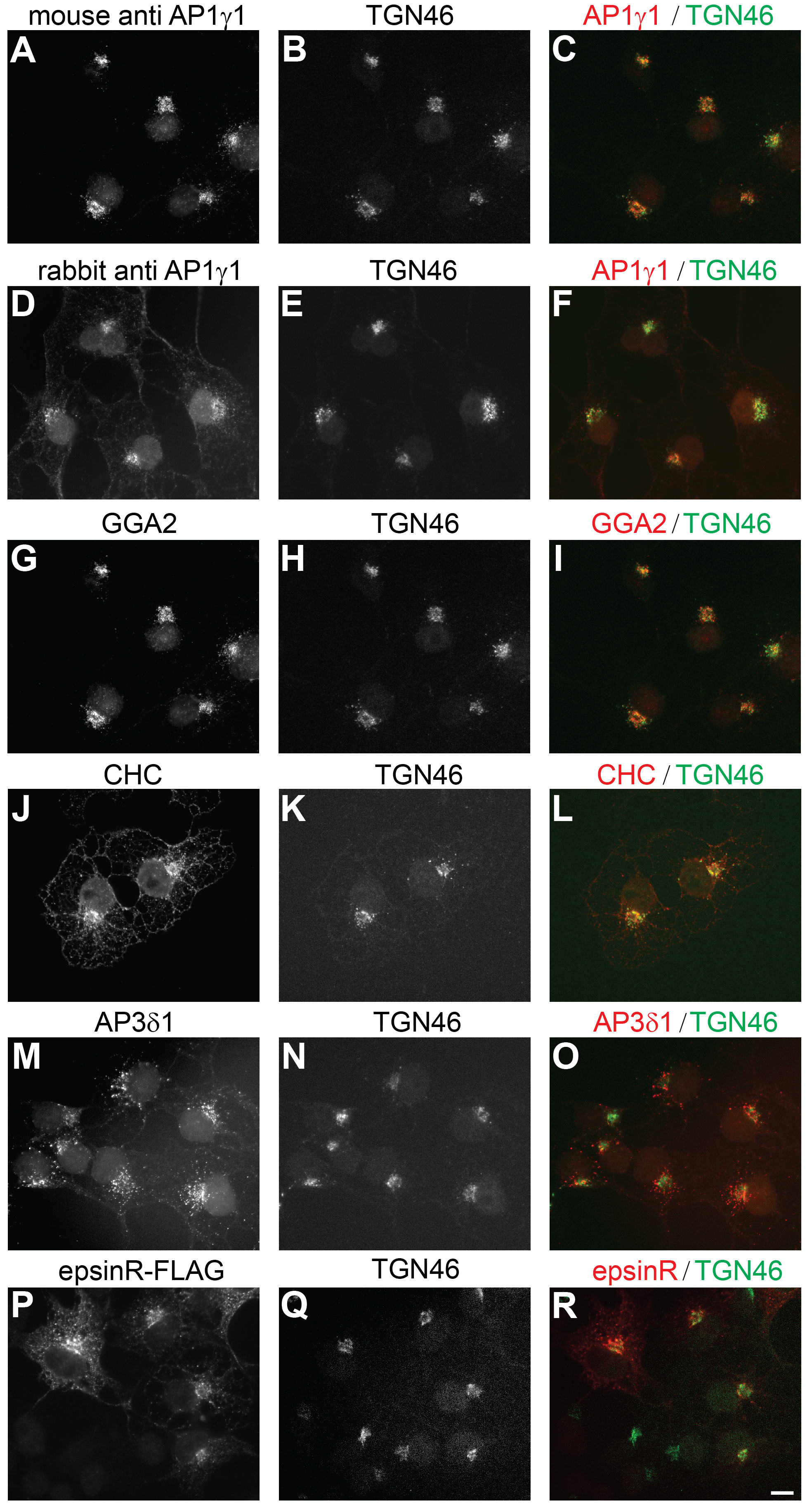

Supplement: Figure S2 — The localization patterns of clathrin and cargo adaptors in COS7 cells. COS7 cells were untransfected (A–O) or transfected with plasmids encoding FLAG-tagged epsinR (P–R). Day 1 after transfection, cells were analyzed by immunofluorescence utilizing the indicated antibodies. Representative fluorescent images were shown. Scale bar, 10 μm. [file Image_2.JPEG]

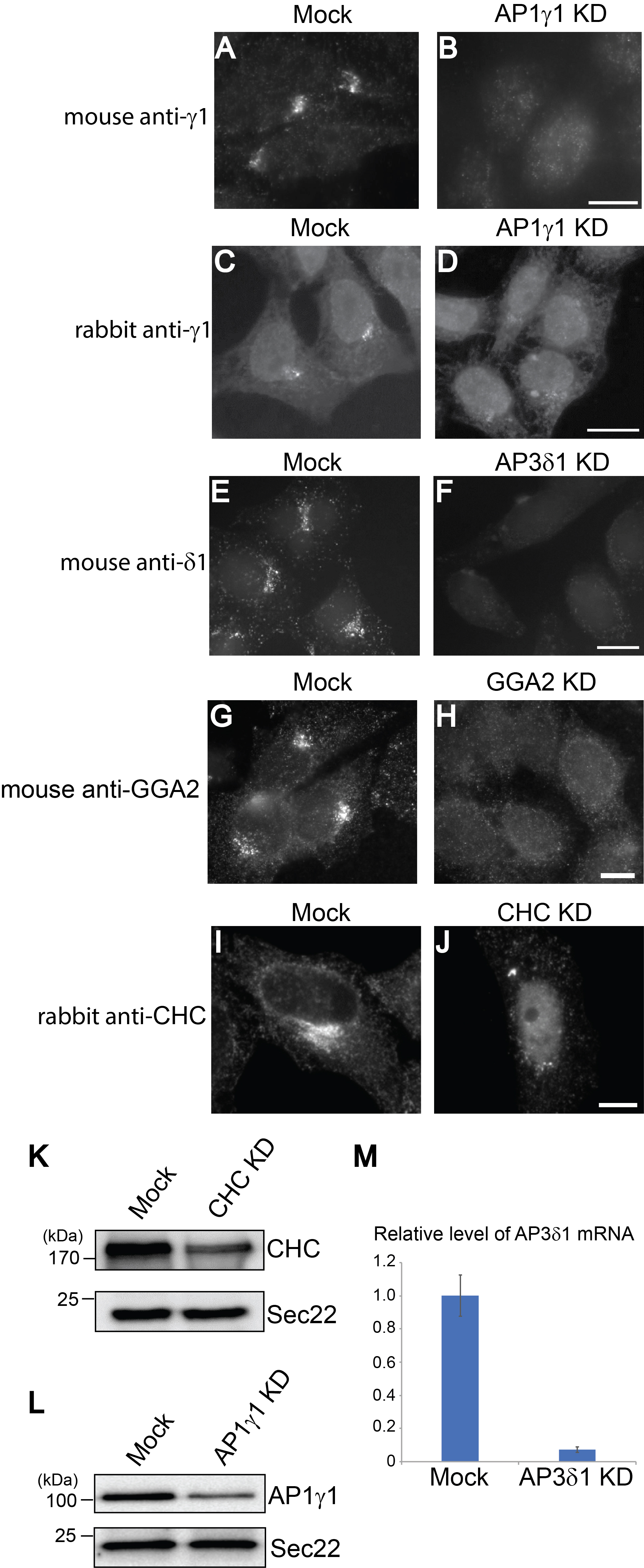

Supplement: Figure S3 — The localization patterns of clathrin and the indicated cargo adaptors in HeLa cells. HeLa cells were transfected with control siRNA (A,C,E,G,I) or transfected with siRNA against the indicated cargo adaptors (B,D,F,H) or clathrin heavy chain (J). Day 3 after transfection, cells were analyzed by immunofluorescence utilizing the indicated antibodies. Representative fluorescent images were shown. Scale bar, 10 μm. (K–M) HeLa cells were mock transfected or transfected with siRNA against AP1γ1, clathrin heavy chain or AP3δ1. On day 3 after transfection, cells were analyzed by immunoblot (K,L) or RT-PCR (M). [file Image_3.JPEG]

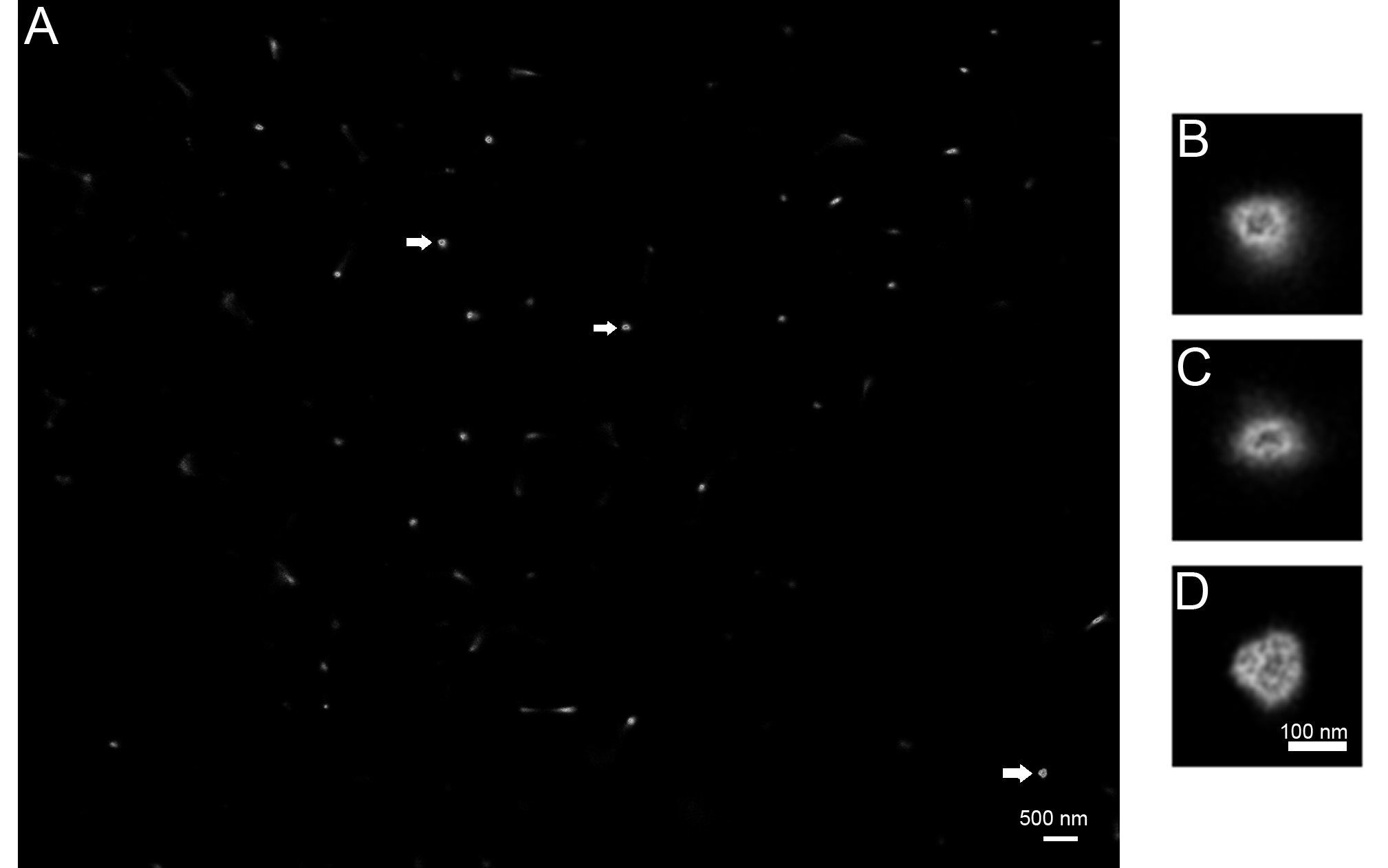

Supplement: Figure S4 — The localization patterns of clathrin heavy chain on the plasma membrane of COS7 cells after stimulating with epidermal growth factor (EGF). (A) COS7 cells were treated with 5 ng/ml EGF for 5 min and then stained with antibodies against clathrin heavy chain. Localization patterns of clathrin heavy chain were analyzed by STORM. Scale Bar, 500 nm. (B–D) Magnified views of the structures highlighted by arrows in (A). Scale Bar, 100 nm. [file Image_4.JPEG]

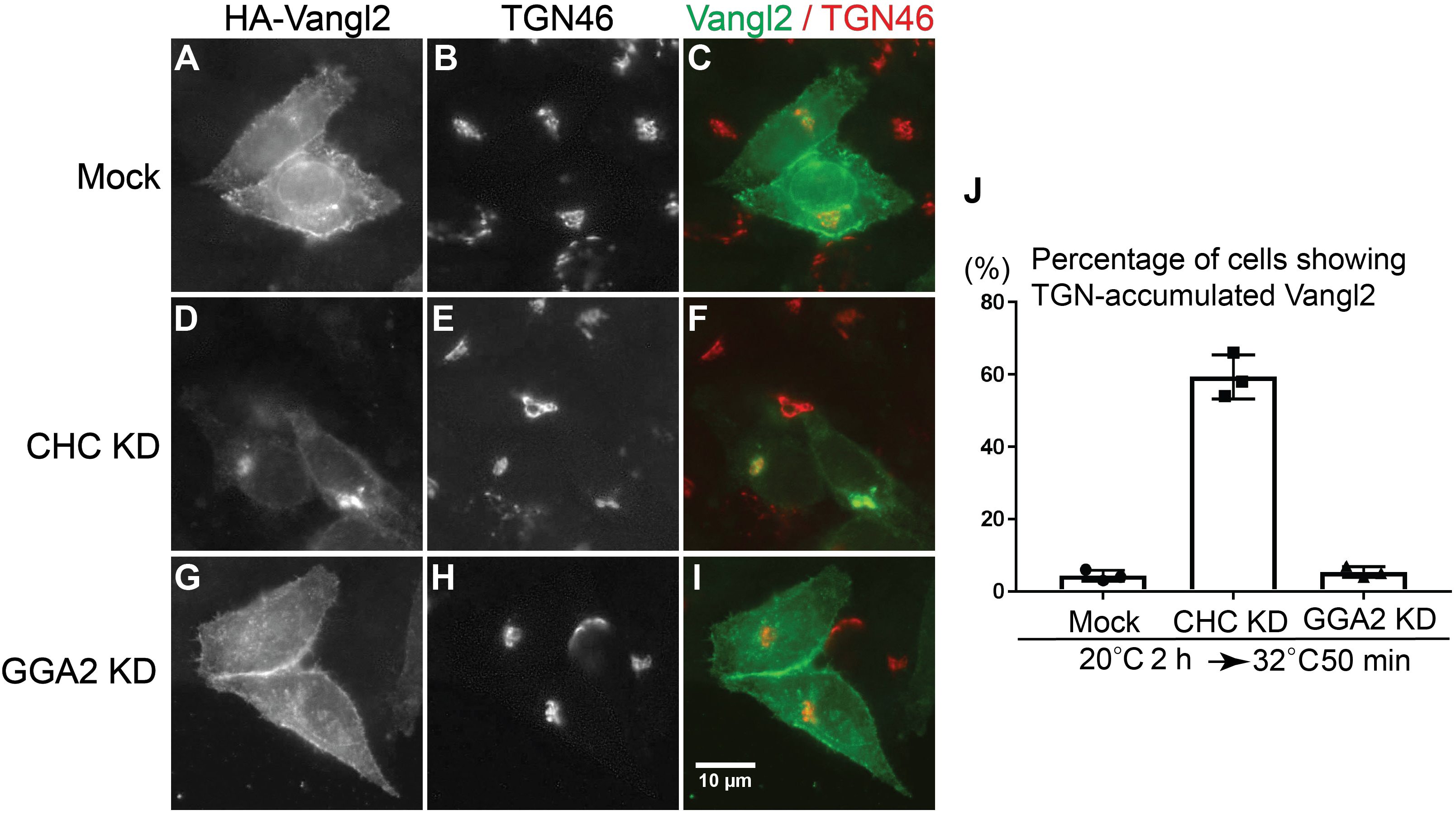

Supplement: Figure S5 — TGN export of Vangl2 is independent of GGA2. (A–I) HeLa cells were mock transfected (A–C) or transfected with siRNA against clathrin heavy chain (CHC, D–F) or transfected with siRNA against GGA2 (G–I) and re-transfected after 48 h with plasmids encoding HA-Vangl2 (A–I). On day 3 after knockdown, cells were incubated at 20°C for 2 h then shifted to 32°C for 50 min in the presence of cycloheximide. After incubation, cells were analyzed by immunofluorescence. Scale bar, 10 μm. (J) Quantification of the percentage of cells showing TGN-accumulated Vangl2 in cells treated with control siRNA or siRNA against CHC or GGA2 after incubation at 32°C (mean ± SD; N = 3; >150 cells counted for each experiment). [file Image_5.JPEG]

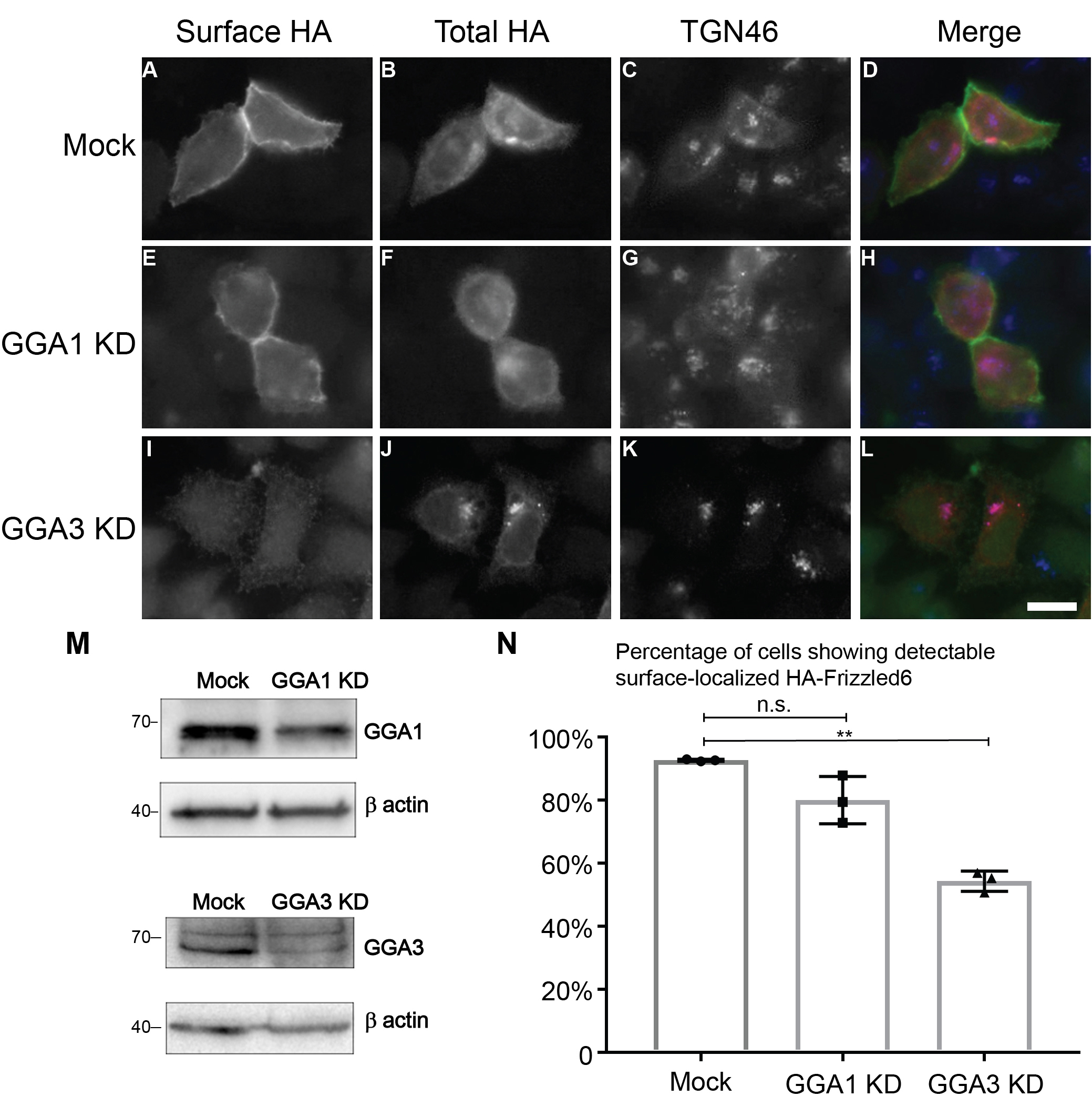

Supplement: Figure S6 — Knockdown of GGA3 but not GGA1 causes defects in surface delivery of Frizzled6. (A–L) HeLa cells were mock transfected (A–D) or transfected with siRNA against GGA1 (E–H) or siRNA against GGA3 (I–L) and re-transfected after 48 h with plasmids encoding HA-Frizzled6. On day 3 after knockdown, cells were incubated at 20°C for 2 h then shifted to 32°C for 50 min in the presence of cycloheximide. After incubation, cells were analyzed by immunofluorescence. The surface-localized HA-Frizzled6 and the total HA-Frizzled6 were stained by mouse and rabbit anti-HA antibodies, respectively. Scale bar, 10 μm. (M) HeLa cells were mock transfected or transfected with siRNA against GGA1 or GGA3. On day 3 after transfection, cells were analyzed by immunoblot. (N) Quantification of the percentage of cells showing detectable surface localized Frizzled6 in cells treated with control siRNA or siRNA against GGA1 or GGA3 after incubation at 32°C (mean ± SD; N = 3; >100 cells counted for each experiment). **p < 0.01 by two-tailed Student's t-test. [file Image_6.JPEG]

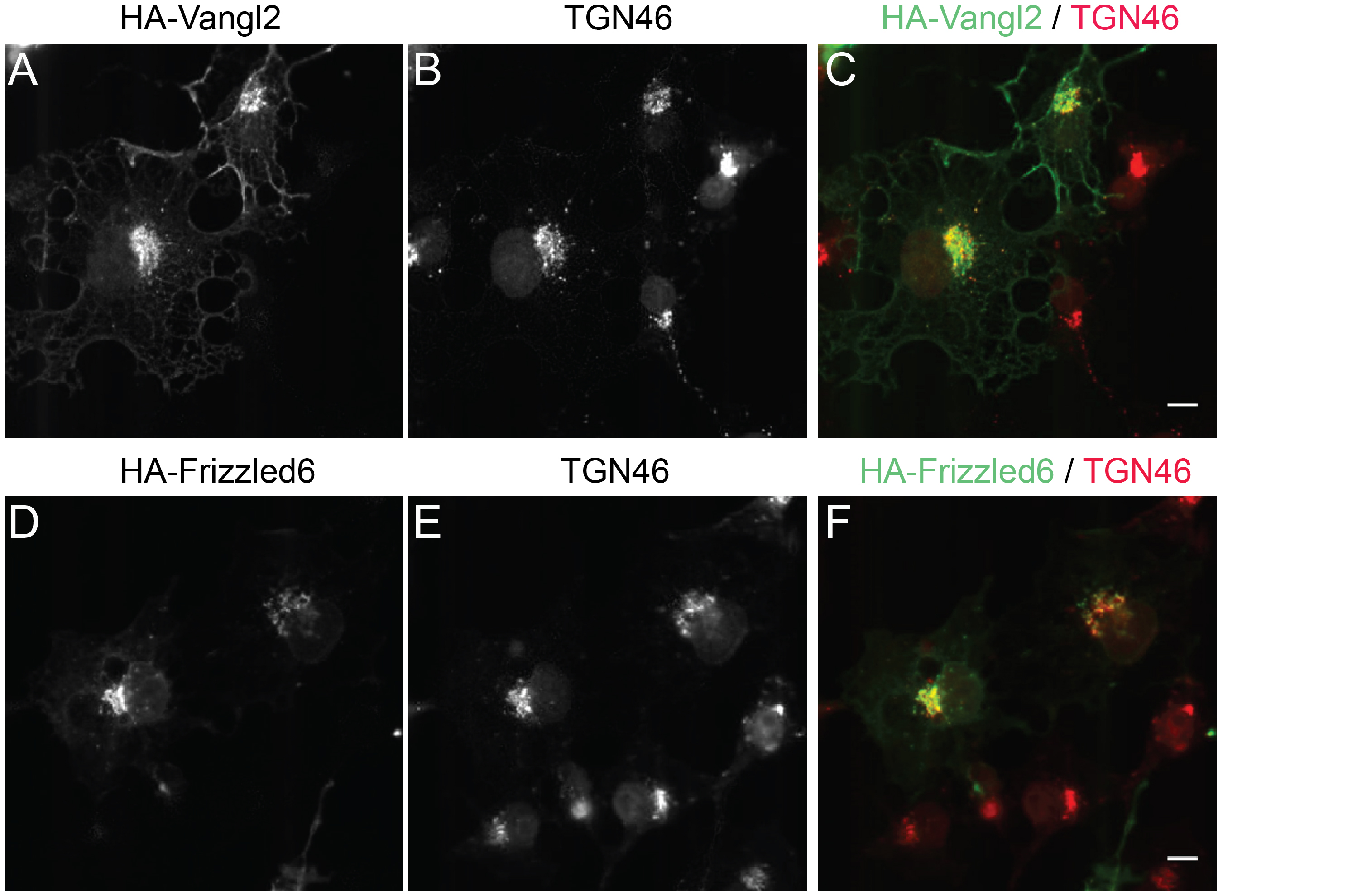

Supplement: Figure S7 — Localizations of Vangl2 and Frizzled6 after temperature shit. (A–F) COS7 cells were transfected with HA-Vangl2 (A–C) or HA-Frizzled6 (D–F). Day 1 after transfection, cells were incubated at 20°C for 2 h and then at 32°C for 5 min. The localizations of Vangl2, Frizzled6, and TGN46 was then analyzed by immunofluorescence. Scale bar, 10 μm. [file Image_7.JPEG]

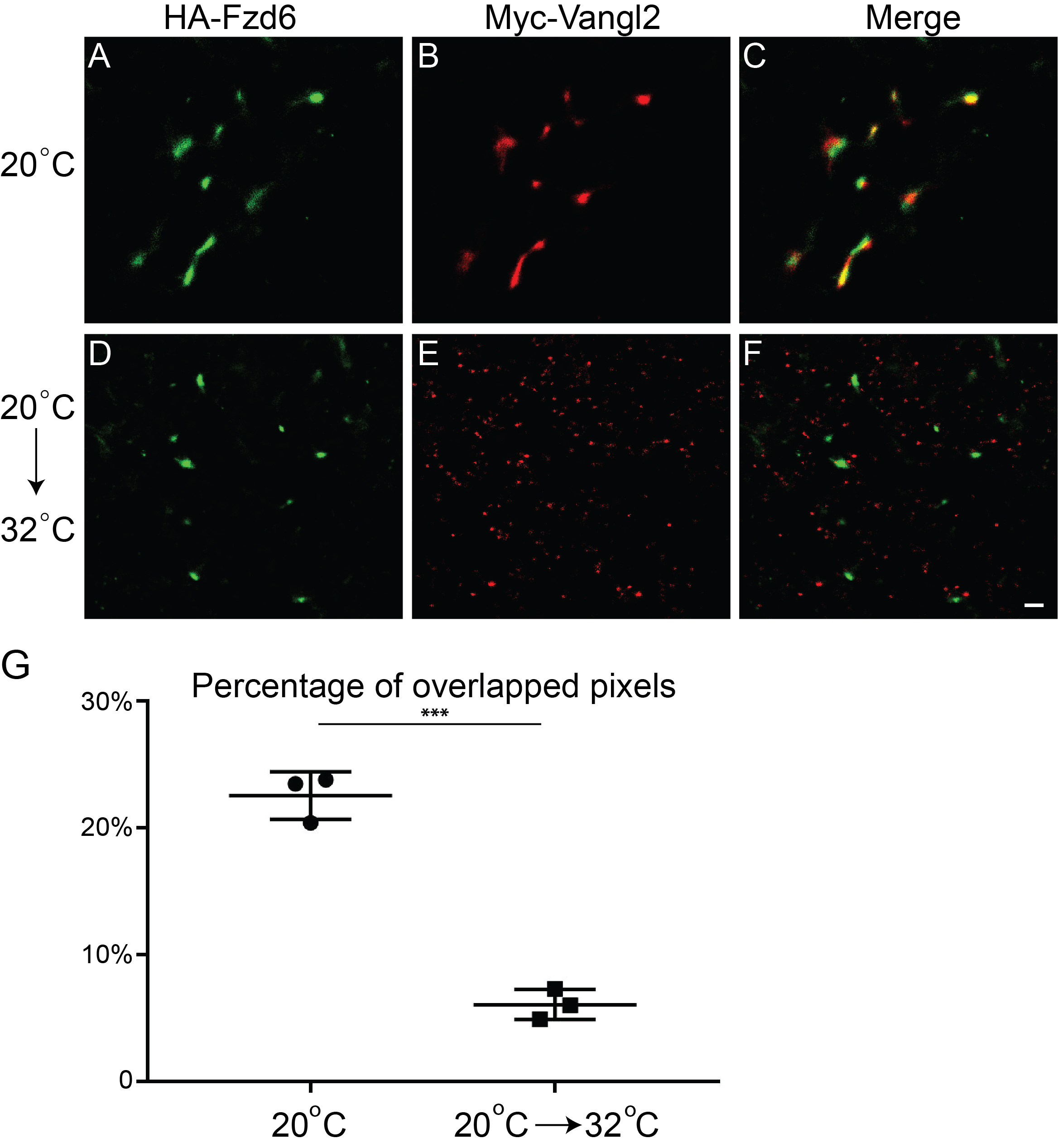

Supplement: Figure S8 — Analysis of the spatial relationships between Vangl2 and Frizzled6 upon exiting the TGN. (A–F) COS7 cells were co-transfected with HA-Frizzled6 and Myc-Vangl2. Day 1 after transfection, cells were incubated at 20°C for 2 h (A–C) or incubated at 20°C for 2 h and then at 32°C for 5 min (D–F). After incubation, cells were stained with antibodies against HA tag and Myc tag. Two-color STORM was then utilized to visualize localizations of Vangl2 and Frizzled6. (G) Quantification of the percentage of overlapped pixels between Vangl2 and Frizzled6 in cells incubated at 20°C for 2 h or in cells incubated at 20°C for 2 h and then at 32°C for 5 min (mean ± SD, based on three super-resolution images each). ***p < 0.001 by two-tailed Student's t-test. [file Image_8.JPEG]

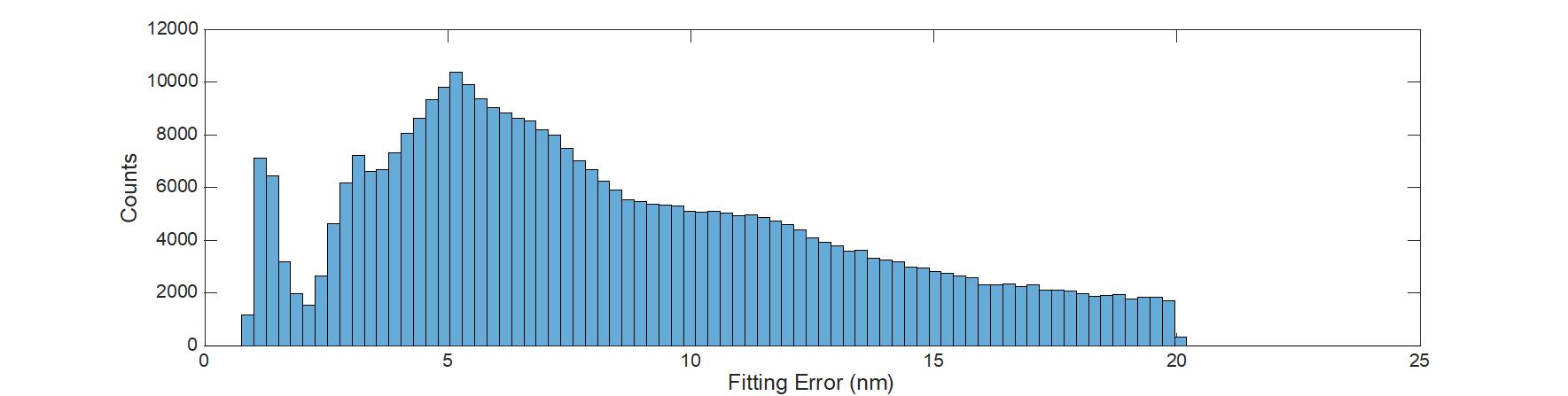

Supplement: Figure S9 — The histogram of the localization error of Figure 5A. The software rejects any fitting with error >20 nm. [file Image_9.JPEG]
